# Metformin as Potential Therapy for High-Grade Glioma

**DOI:** 10.3390/cancers12010210

**Published:** 2020-01-15

**Authors:** Marek Mazurek, Jakub Litak, Piotr Kamieniak, Bartłomiej Kulesza, Katarzyna Jonak, Jacek Baj, Cezary Grochowski

**Affiliations:** 1 Department of Neurosurgery and Pediatric Neurosurgery, Medical University of Lublin, Jaczewskiego 8, 20-954 Lublin, Poland; marekmazurek@hotmail.com (M.M.); jakub.litak@gmail.com (J.L.); pkamieniak@poczta.onet.pl (P.K.); kuleszabartek88@gmail.com (B.K.); 2 Department of Immunology, Medical University of Lublin, Jaczewskiego 8, 20-954 Lublin, Poland; 3 Department of Foregin Languages, Medical University of Lublin, Jaczewskiego 4, 20-090 Lublin, Poland; kasia.jonak@gmail.com; 4 Department of Anatomy, Medical University of Lublin, Jaczewskiego 4, 20-090 Lublin, Poland; jacek.baj@me.com

**Keywords:** glioblastoma multiforme, GBM, metformin, clinical trial, glioma

## Abstract

Metformin (MET), 1,1-dimethylbiguanide hydrochloride, is a biguanide drug used as the first-line medication in the treatment of type 2 diabetes. The recent years have brought many observations showing metformin in its new role. The drug, commonly used in the therapy of diabetes, may also find application in the therapy of a vast variety of tumors. Its effectiveness has been demonstrated in colon, breast, prostate, pancreatic cancer, leukemia, melanoma, lung and endometrial carcinoma, as well as in gliomas. This is especially important in light of the poor options offered to patients in the case of high-grade gliomas, which include glioblastoma (GBM). A thorough understanding of the mechanism of action of metformin can make it possible to discover new drugs that could be used in neoplasm therapy.

## 1. Introduction

Gliomas are the most common primary neoplasms of the central nervous system (CNS) and they constitute approximately 30–40% of these types of cancers [1,2]. In the case of malignant tumors, this percentage is even higher and amounts to as much as 80% [2,3]. Gliomas are derived from glial cells or glial precursor cells [4]. For their proper description, the WHO (World Health Organization) created the Classification of Tumors of the Central Nervous System, dividing them into four groups in terms of malignancy [5,6]. High-grade gliomas (WHO grade III and IV gliomas) account for the vast majority of all primary tumors of the CNS. The most common and aggressive form of glioma is glioblastoma multiforme (GBM, WHO grade IV glioma). It is estimated that it constitutes 15% of diagnoses [7]. Despite surgery, radiotherapy, and temozolomide chemotherapy (the main treatments for gliomas), the mean overall survival is about 14.6 months [8,9,10].

Metformin (MET), 1,1-dimethylbiguanide hydrochloride, is a biguanide drug that is used as the first-line medication in the treatment of type 2 diabetes. It suppresses gluconeogenesis in the liver, sensitizes peripheral cells to insulin, increases glucose uptake, inhibits mitochondrial respiration, and reduces glucose absorption by the gastrointestinal tract. The last of these functions is responsible for the majority of side effects [11,12,13,14]. Metformin is a safe drug; it has had a long history of use and is used by millions of patients on a daily basis. Research suggests that metformin is not only a relatively safe drug in the non-diabetic patients’ group, but may also be associated with positive effects on the body such as weight loss or reduced cardiovascular risk [15,16,17]. Moreover, its regular use contributes to a decrease in the likelihood of stroke in patients with type 2 diabetes. It has also been proved to reduce mortality associated with cardiovascular disease [18,19]. The most dangerous complication of metformin is lactic acidosis; however, it rarely occurs in patients [20,21,22]. What is more, the majority of patients in whom it had developed had a history of independent risk factors for this condition [23]. However, no studies have yet been conducted to determine the exact risk of lactic and keto acidosis following the administration of the drug in a patient population with normal carbohydrate metabolism. Ongoing observations of the effect of metformin on non-diabetic individuals are presently at the recruitment stage (NCT03772964).

Currently, metformin is one of the most common oral anti-diabetic drugs registered for clinical use. It is widely used due to its relative safety, anti-hyperglycaemic activity, and bodyweight reduction influence [24]. Recently, its potential influence on the pathogenesis of tumors has also been observed [25,26,27]. The use of metformin has been associated with better overall and progression-free survival of patients with high-grade glioma [9]. Repurposing metformin as cancer treatment is already being tested in a range of clinical trials for a variety of cancers. The aim of the present study is to present a review of the literature on metformin as a potential treatment drug in high-grade gliomas (Table 1).

## 2. The Effect of Metformin on the Course of Cancer

Carbohydrate disorders pose a particularly serious issue in modern medicine. According to forecasts, by 2030, 439 million adults worldwide will have struggled with the problem of diabetes [28]. These disorders also affect the occurrence of tumors. Chaichana et al., examined 182 patients with low-grade gliomas (WHO grade II) for the effect of persistent hyperglycaemia on treatment outcomes. They have shown that it results in a decrease in patient survival and the increase in the frequency of relapses [29,30]. Similar results have been observed with high-grade gliomas as well as in studies conducted precisely on patients with GBM [30,31,32].

Type 2 diabetes, as well as obesity, has been identified as an independent factor of poor prognosis in patients with high-grade gliomas [31]. This was also confirmed by studies carried out by Welch et al. They showed that, among patients suffering from GBM, the prognosis is worse in the presence of diabetes [33]. This indicates the possible potential role of drugs that lower blood glucose in glioma therapy. It is worth noting that anti-cancer treatment itself can affect carbohydrate metabolism. Steroids, including dexamethasone, are the primary medicines for preventing brain edema due to the presence of a tumor. One side effect of their use is hyperglycemia. In their observations of patients with newly diagnosed GBM, Derr et al., also confirmed the negative impact of high glucose values on patients’ prognosis. At the same time, they drew attention to the fact that proper control of the doses of steroids taken makes it possible to limit the severity of hiperglycemia, thus contributing to the improvement of the clinical outcomes of patients [34]. This shows how important it is to know exactly the pathomechanism of the cancer process as it allows physicians to effectively plan oncological treatment.

As mentioned before, metformin is among the drugs that lower blood glucose. The potential effect of the activity of metformin has been described in the pathogenesis of many cancers. Inhibition of tumor cells growth after metformin administration was observed, among others, in colon, breast, prostate, pancreatic cancer, leukemia, melanoma, lung, and endometrial carcinoma [6,35,36,37,38,39,40,41,42,43,44,45,46,47]. This effect was visible in both in vitro and in vivo experiments. Similar observations have been made for gliomas [48,49]. This applies to the inhibition of tumor cell proliferation as well as the inhibition of their differentiation and invasiveness [35,39,50,51,52,53,54,55]. It can also lead to the death of GBM cells as the result of apoptosis or autophagy [35,39,51]. The use of metformin may also increase the effectiveness of standard glioma therapies [50,51,56]. In studies conducted by Adeberg et al., on a cohort of 276 patients with primary GBM, longer progression-free survival was demonstrated in diabetic patients treated with metformin [57]. This observation included only patients with GBM. Studies by Seliger et al., concerned 1093 patients with high-grade gliomas (also WHO III). Additionally, in this case, improved overall and progression-free survival in patients treated with metformin has been shown. Interestingly, this relationship was only relevant to grade III gliomas. For grade IV, no relationship was found between metformin intake and the patients’ life expectancy [9]. Similar results were obtained from another analysis also carried out by Seliger et al., In this case, they studied the effect of metformin use on 1731 patients with GBM. Similarly, no significant relationship between the use of the drug as monotherapy and overall survival and progression-free survival has been demonstrated [58]. The discrepancy of data from various experiments indicates the need for further observation on this issue.

Some researchers also indicate the potential for also using metformin in cancer prevention [59,60,61,62]. It has been noticed that, in patients with diabetes on long-term treatment with this drug, the chance of developing neoplasm is lower compared to the controls [63]. However, the analysis by Seliger et al., showed no significant correlation between the occurrence of the disease and the earlier use of metformin in patients with gliomas [64]. However, confirming this issue requires further research.

Metformin has a number of physicochemical characteristics that would promote its use in the treatment of brain tumors if its effectiveness were confirmed. The drug particles are characterized by their small size and amphoteric character, thus determining their hydrophilicity and high solubility in water. However, it also has a non-polar hydrocarbon chain, giving it its lipophilic properties. This makes it possible for metformin to bind to the lipid domains of cell membranes [65]. The ability of the drug to cross the blood-brain barrier has been demonstrated in studies conducted by Łabuzek et al., on a rat model. The authors checked how the oral administration of metformin changes its concentration in various regions of the brain, such as the frontal cortex, the olfactory bulb, the hypothalamus, the striatum, the pituitary gland, and the cerebellum. They showed a high ability of metformin to cross the blood-brain barrier and redistribution in the central nervous system [66]. Convergent results were also noted in observations of other authors [66,67,68]. This is an important property in the case of high-grade glioma because, due to its location, effective action within them requires overcoming the barrier, which is not achieved by many other drugs. The widespread use of it in the world is also of great importance. Thanks to this, it has already been thoroughly tested for side effects and potential interactions with other drugs. This can potentially significantly reduce the time needed for the official implementation of metformin in standard patient therapy.

## 3. Variety of High-Grade Glioma and Cancer Treatment

Accurate presentation of the effect of metformin on the pathogenesis of gliomas requires presenting the complexity of the process of gliomogenesis. This group of cancers is characterized by very high molecular and epigenetic diversity. After analyzing these differences, the above division into high-grade gliomas and low-grade gliomas seems to be artificial and does not fully reflect the characteristics of these tumors. Genomic abnormalities driving tumorigenesis were classified based on a large multicentre analysis of The Cancer Genome Atlas (TCGA) Research Network. Verhaak et al., by consolidating data on 206 patients with GBM samples, created a classification, distinguishing four basic GBM subtypes: classical, neural, proneural, and mesenchymal. The classical subtype was mainly characterized by the epidermal growth factor receptor (EGFR) alterations. It is also worth noting that in this group of gliomas there were no mutations in the TP53 suppressor gene whose overall prevalence in GBM is very common. The mesenchymal subtype was dominated by disorders associated with the neurofibromin 1 (NF1) gene involved in the negative regulation of the RAS/MAPK pathway. Abnormalities in PTEN and NF-κB, RELB, TRADD, and TNFRSF1A pathways were also characteristic. The neural type was distinguished by an increased expression of neuronal markers: GABRA1, SLC12A5, SYT1, and NEFL. The last type of GBM called proneural was associated with more frequent occurrence of TP53 mutation and the amplification of PDGFRA (platelet-derived growth factor receptor A) and IDH1 (Isocitrate dehydrogenase 1) [69,70]. A similar classification was also proposed by Sturm et al. Based on global methylation patterns, they distinguished six subtypes of GBM. In their classification, they enriched the previously existing division created on the basis of TCGA with subtypes associated with mutations in the H3F3A or IDH1 range. This is of great importance, especially in the youngest age group of patients, because it is estimated that H3F3A or IDH1 disorders affect about 30–40% of these cases [71]. The IDH (isocitrate dehydrogenase) gene codes an enzyme involved in the oxidative decarboxylation of isocitrate, producing α-ketoglutarate [72]. The presence of mutations in one of its isoforms (IDH1) has been demonstrated for many cancers of the glioma group: Oligodendroglioma, astrocytoma, and glioblastoma multiforme. In the case of GBM, this mainly concerned the secondary subtype, developing from less malignant forms of cancer [73,74,75]. IDH1 mutations result in the transformation of α-ketoglutarate to (D) -2-hydroxyglutarate, the accumulation of which leads to a disruption of the function of α-ketoglutarate-dependent enzymes resulting in hypermethylation of DNA and histones [76,77]. This inhibits the activity of suppressor genes and activates oncogenes, inducing tumorigenesis [78]. Ceccereli et al., also looked into the IDH mutation status in gliomas. In their analysis, they associated the glioma subtype characterized by the IDH mutation with an unfavorable prognosis and DNA methylation, while in the IDH-wild type subtype, the prognosis was more optimistic [79].

In the aforementioned paper, Seliger et al., suggested a relationship between the IDH status of the tumor and the sensitivity of cancer cells to metformin. In their observations, the effect was to improve the survival of patients with WHO III glioma in which IDH mutations are common in the absence of such effect in patients with WHO IV glioma where mutant IDH is less common [9]. This relationship was probably conditioned by glutamine metabolism. Cells with mutated IDH are characterized by glutamine-related metabolic vulnerability due to the existence of deficits in the reductive glutamine anaplerosis. In turn, metformin can interfere with the anaplerotic entry of glutamine into the tricarboxylic acid cycle by inhibiting the oxidative glutamine anaplerosis. As a result, the imposition of these two conditions can lead to disorders of cell metabolism, reducing their survival [80,81,82].

The above data show a variety of gliomas and highlight the multitude of metabolic pathways, in which gliomogenesis can take place, forming a network of interrelationships. In light of these facts, creating one universal drug that could be used to treat all patients with high-grade glioma seems impossible. Understanding this issue is necessary before analyzing the effect of metformin on cancer pathogenesis. Due to its biochemical properties, metformin may exhibit anti-tumor properties against some cell lines, while against others this effect may not be noticeable. Thorough understanding of the molecular profile of glioma may determine the validity of using metformin in the therapy of a particular patient. The relationships between the activity of individual metabolic pathways of malignant gliomas and their resistance to treatment are described in the work of Stupp et al., The authors analyzed the role of EGFR, PKC, and Ras in the induction of chemo and radio resistance. At the same time, MGMT activities were assigned the greatest role in forming this phenomenon. This mechanism is described in detail in the further part of the article [83]. The course of the disease and the sensitivity of cells to treatment depend not only on the molecular type of glioma, but also on many other factors that can modify the tumor environment by affecting its phenotype. These include, among others, hypoxia or the cell immune response system [84].

## 4. Metformin: Antineoplastic Mechanism

**Figure 1:**
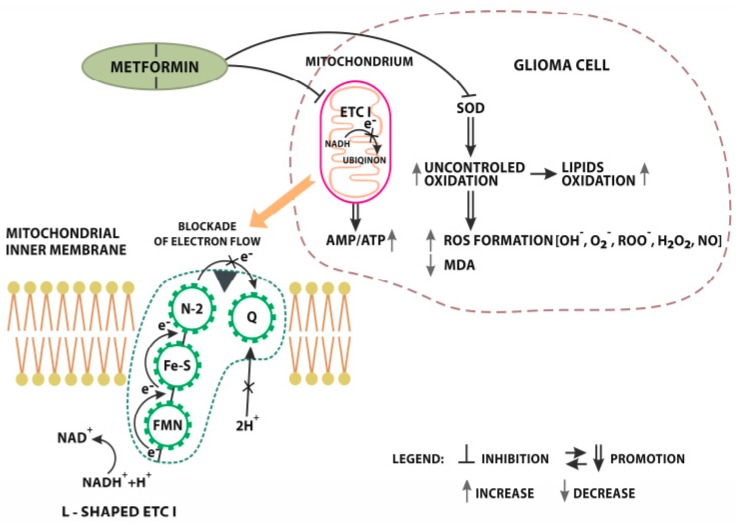
Metformin AMPK independent signaling. Inhibition of L-shaped electron transport chain complex I (ETC I) localized on mitochondrial inner membrane. NADH + H transfer electrons to FMN (flavin mononucleotide) preluding further reduction to FMNH2). In the next step, electrons move along iron-sulfur groups to N2 (Iron sulphur protein) where ETC1 uses this electrical work to pump H+ ions out of the matrix. Electrons are finally delivered from the Iron sulfur complex to Q (Ubiquinone). After the acceptance of electrons, ubiquinone uptakes two protons from the matrix. The whole process is finished with a full transformation into a reduced form of ubiquinol-quinol QH2. Metformin blocks electron flow from the Iron sulphur complex to ubiqinon. This blockade results in significant reduction of proton pomp efficiency and the growth of the AMP/ATP ratio. Metformin influences oxidation processes. MET directly limits SOD (Superoxide dismutase) activity. Inhibition of SOD precedes the uncontrolled oxidation of lipids and excessive ROS (Radical Oxygen Species) formation [OH^−^-hydroxyl radical, O_2_^−^-superoxide anion, ROO-peroxyl radical, H_2_O_2_ hydrogenperoxide_,_ NO nitric oxide.].

To this day, the complete mechanism of action of metformin on cancer cells has not been known. As mentioned earlier, it causes the inhibition of tumor cell proliferation and a decrease in the rate of cancer development, but the exact path leading to this effect is still a mystery [85]. One of the reasons for this is the multidirectional signaling pathways stimulated by the presence of drug particles. Metformin can act both through adenosine monophosphate kinase protein (AMPK: dependent mechanism) and without AMPK (AMPK-independent) (Figure 1) [11,13].

At present, two main points of drug anti-tumor activity are indicated:

- acting on mitochondria through oxidative stress, and
- acting by regulating AMPK pathway activity [86,87].

### 4.1. Oxidative Stress

One of the potential mechanisms of action of metformin is the impact on the production of ATP and oxygen consumption [50]. It has been shown that the target of this drug may be the electron transport chain complex (ETC) present in the mitochondria [88]. It consists of a number of complexes whose role is to transfer electrons from donors to acceptors through a redox reaction. At the same time, H+ ions’ transmembrane transport takes place, resulting in a proton gradient. This enables the production of adenosine triphosphate (ATP) molecules [89].

The ability of metformin to inhibit one of the ETC components, which is the electron transport chain complex I (ETCI), has been described. In the case of Owen et al., the experiments were conducted on an animal model [88,90]. ETCI is the largest component of ETC. Its role is to transfer electrons from the NADH matrix to ubiquinone [91]. ETCI inhibition results in a change in calcium ion levels and mitochondrial transmembrane potential levels. The AMP/ATP ratio is also disturbed, leading to an increase in oxidative stress, which affects cells [88,92,93]

These changes also affect the metabolism of both the cell and the whole body. This leads to the predominance of the catabolic processes and mitochondrial biogenesis, while inhibiting the production of proteins and anabolic processes. This phenomenon was observed in the studies on GBM cell lines by Sesen et al., In all the GBM lines tested, ETCI activity was significantly reduced after metformin administration. The authors stated that the drug decreases mitochondrial oxygen consumption as well as causes an increase in lactate and glycolytic ATP production [50]. It is worth noting that these effects may not be visible in all subtypes of glioma. This is associated with the expression of NADH-ubiquinone reductase (NDL1). Wheaton et al., showed that it has the ability to reverse the effect of metformin on the mitochondrial complex I [94]. Similar conclusions were made by Wu et al., in their work on inhibiting the progression of Head and Neck Squamous Cell Carcinoma (HNSCC) by metformin. They showed that NDI1 expression blocked metformin’s ability to inhibit mitochondrial complex 1, activated AMPK, and reduced mTOR pathway signaling, resulting in a lack of effective inhibition of tumor development. The above phenomenon occurred in both in vitro and in vivo observations [95].

Metformin can also affect oxidative stress by acting on superoxide dismutase (SOD) activity. The role of SOD is to protect cells against the effects of free oxygen radicals. It removes free radicals, which reduces their effect on tissues [96]. The research of Xiong et al., has shown that metformin can reduce SOD activity content in GBM cells, resulting in an increase in their exposure to free radicals and their damage. They also noted a decrease in malondialdehyde (MDA) levels, which is a good indicator of oxidative stress in the cell [85]. 

### 4.2. Metformin and Adenosine Monophosphate-Activated Protein Kinase (AMPK)

**Figure 2:**
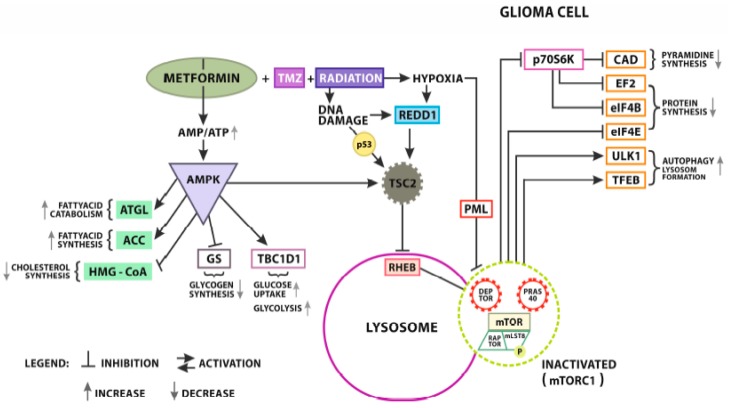
Metformin (MET) AMPK dependent signaling. An increase of AMP/ATP ratio activates AMPK/TSC2 signaling. TSC2 (Tuberous Sclerosis Complex 2) activation results in RHEB- mTORC 1 complex inhibition (Ras homologue enriched in the brain). Another signaling pathway causing mTORC 1 inhibition is stimulated by hypoxia and DNA damage as a result of MET + Temozolomide (TMZ) and radiation synergistic effect. Hypoxia also inhibits indirectly mTORC1 by PML (promielocitic leukemia protein) activation. The right side of the scheme presents the effects of mTORC1 inhibition in the glioma cell. Inactivated mTORC1 reduces p70S6K (Ribosomal protein S6 kinase) activity resulting in the decrease of CAD (trifunctional multi-domain: carbamoyl-phosphate synthetase 2, aspartate transcarbamylase, and dihydroorotase). Reduced CAD limits pirimidine synthesis in GBM cells. Collaterally, the reduction of p70S6K activity has a negative influence on EF2 (Elongation factor 2) andeIF4B (Eukaryotic translation initiation factor 4B) leading to limited protein synthesis. Contrary, the deactivation of mTORC1 promotes ULK-1(autophagy activating kinase) and TFEB (Transcription factor EB) proautophagic factors supporting new lysosomes formation. The left side of scheme presents AMPK’s direct effect on lipids and glucose metabolism in GBM. Concerning lipids, AMPK promotes ATGL (Adipose triglyceride lipase) activity resulting in fatty acids’ catabolism, and regulates ACC’s (Acetyl-CoA carboxylase) activity in fatty acids synthesis. Additionally, it reduces HMG-CoA’s (HMG-CoA reductase) activity resulting in the reduction of cholesterol synthesis. As far as glucose metabolism is concerned, AMPK inhibits glycogen formation limiting GS (glycogenesis), promoting TBC1D1 (TBC1 domain family member 1) and it increases glucose uptake and glycolysis. To sum up, AMPK inhibits mTOR and improves metabolic reprogramming, which consequently suppresses tumor growth.

Participation in the regulation of AMP-activated protein kinase (AMPK) may be a very important point of action of metformin. It is a protein classified as serine/threonine kinase. Physiologically, it plays a role in regulating enzymatic changes in fat metabolism and cholesterol synthesis (Figure 2).

However, it has been found that AMPK dysfunction may be associated with the development of a neoplasm process. Chhipa et al., in their work on human primary GBM lines and xenograft mice, have shown that cancer-associated chronic stress increases the activity of the AMPK pathway. The authors also emphasized the importance of GABPA and HIF1α transcription factors in the process [97]. The mechanism of cellular stress in GBM cells has also been studied by Zhang et al., In this case, the authors focused on the role of phosphoinositide 3-kinase enhancer-activating Akt (PIKE-A), which is a pro-oncogenic factor that promotes cancer cell proliferation and tumor growth. Researchers have shown that AMPK may participate in PIKE-A phosphorylation, thereby inhibiting the development of the cancer process. This was done indirectly by disrupting the function of CDK4 and inhibiting the Rb pathway [98]. Similar disorders were also shown in the works of other researchers and were carried out on both cell lines and animal models [99,100,101]. This shows how important it is to fully understand the complexity of this route and to potentially control its course.

The level of its activity is regulated by the ratio of AMP (adenosine monophosphate) or ADP (adenosine diphosphate) to ATP (adenosine triphosphate). Thanks to this, it plays the role of a specific energy level sensor in the cell. Activation of AMPK takes place through phosphorylation at T172 of the α-subunit. AMPK activity is regulated by the action of phosphatases reversing this process. This is done through the competitive binding of AMP, ADP, or ATP to its γ-subunit. AMP or ADP binding blocks access phosphatases to residue T172 of AMPK’s α-subunit, allowing it to remain active. In turn, the presence of ATP competitively displaces AMP and ADP, influencing the decrease in AMPK activity [102]. The decrease in ATP levels in cells causes an excess of AMP and shift metabolism in the catabolic mode. This leads to increased glycolysis and fatty acid oxidation and gluconeogenesis and the inhibition of protein synthesis and lipid [100].

Another role of the AMPK pathway is the regulation of cell proliferation, migration, and apoptosis [39,103]. As previously mentioned, AMPK activation is induced by an increase in the AMP/APT ratio, indicating a decrease in the level of energy stored in the cell. Its effect is switching cells in a low-power mode, as well as the inhibition of energy intensive processes such as biosynthesis of cell proteins or proliferation [104]. These are processes necessary for the development of neoplasm.

**Figure 3:**
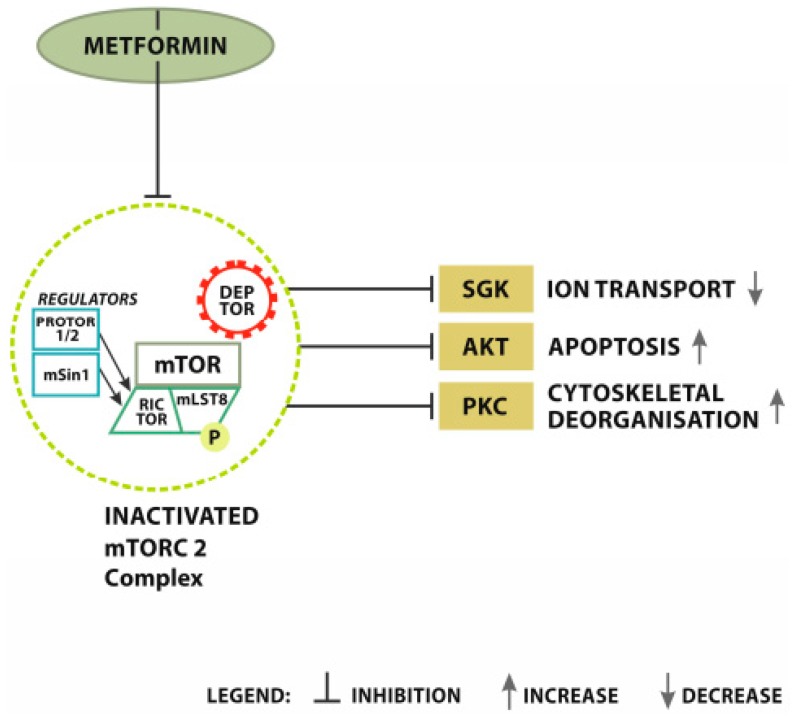
Metformin signaling-mTORC2 inhibition. MET inhibits mTORC2 directly. Inactivated complex limits further typical signaling. Transference restrictive signals from MET on mTORC2 result in the inhibition of SGK (serum and glucocorticoid-induced protein kinase), AKT (serine/threonine-protein kinase), and PKC (protein kinase C) related pathways. MET induced inactivation of mTORC2 results in SGK inhibition. SGK limitation significantly reduces ion transport. AKT inhibition consequently leads to apoptosis and PKC restriction accelerates cytoskeletal deorganisation.

Activation of AMPK leads to phosphorylation of acetyl CoA carboxylase (ACCα) causing blockage of fatty acid (FA) synthesis [105]. Phosphate residues are also attached to TSC2 and RAPTOR leading to blocking of the mammalian target of rapamycin (mTOR) [106,107]. AMPK inhibits mTOR and improves metabolic reprograming, which consequently suppresses tumor growth [108,109]. TOR impacts cellular growth and proliferation through protein synthesis regulation, lipid synthesis cell survival, cell motility, autophagy, and transcription [110]. It controls protein synthesis in downstream pathways as part of mTOR complex 1 and 2. These protein complexes both use mTOR as a core. MTOR complex 1 induces protein, nucleotide, and lipid synthesis, and inhibits autophagy leading, consequently, to cell growth, whereas mTOR complex 2 induces cell proliferation [111,112] (Figure 3).

Activation of AMPK is also involved in cell autophagy. It is a catabolic process whose purpose is to remove unnecessary or dysfunctional components through the controlled distribution of organelles and parts of the cell by itself [113]. Autophagy has been shown to be the major anti-tumor mechanism of metformin use in leukemia and melanoma [114,115]. It occurs through both direct and indirect inhibition of ULK1. It has been observed that AMPK can directly activate ULK1 through its phosphorylation as well as indirectly through mTOR inhibition [113,116]. However, some studies suggest that autophagy induced by metformin may also occur through a mechanism independent of AMPK [114,117].

The possible antitumor effect of metformin in the form of inhibiting the proliferation, invasiveness, and migration of cancer cells by regulating the AMPK/mTOR pathway has already been observed in pancreatic cancer and lung cancer [118,119]. A similar tendency was noticed by researchers in the case of a tumor in the stomach, liver, and nasopharynx [39,50,103,120]. Research conducted by Xiong et al., focused on observing the effect of metformin on AMPK activity in gliomas. They have shown that metformin causes an increase in AMPK expression and a decrease in mTOR protein expression, thereby inhibiting proliferation and increasing tumor cell apoptosis [85]. This seems to confirm the role of AMPK/mTOR signaling pathway in the mechanism of the action of metformin, indicating its potential use in anti-cancer therapy.

However, the involvement of the AMPK pathway in metformin is still a contentious issue. Some researchers suggest that it affects the mTOR protein without AMPK mediation. In studies conducted by Liu et al., the effects of two AMPK agonists, metformin and AICAR, were studied. It was observed that glioma cells (both mouse and human) were characterized by constitutively AMPK activity, while tumor growth was inhibited. This suggests the existence of anti-cancer mechanisms independent of this enzyme [100]. Similar conclusions have been drawn when observing the effect of metformin in inhibiting the development of lung cancer [121].

### 4.3. Influence on REDD1

Another axis of signaling pathways that metformin can affect is the expression of the REDD1 protein (regulated in development and DNA damage response 1), also known as DDIT4 (DNA-damage-inducible transcript 4). It participates in the regulation of cellular response to hypoxia [122]. REDD1 belongs to the negative modulators of the mammalian target of rapamycin (mTOR), constituting a key element regulating its hypoxia-induced signaling pathway [123]. Decline in its expression may lead to a decrease of hypoxia ability to inhibit mTOR, thereby affecting the pace of development of the cancer. Cells in which REDD1 activity is disturbed lose the ability to lower S6K and 4E-BP1 (two major mTOR substrates) phosphorylation following energy depletion [124]. The significance of REDD1 in tumorigenesis has also been proved in the case of GBM [125].

In turn, high levels of REDD1 expression can potentially inhibit mTOR activity. This happens in a situation of cellular stress. Such a phenomenon was observed in the cases of: Starvation, high cell density, decrease in ATP level, as well as under the influence of glucocorticoid treatment and reactive oxygen species [126,127,128]. However, recent data prove that metformin may also induce a similar effect. This correlation was suggested in the case of prostate cancer cells in observations made by Ben Sahra et al. [129]. They noticed an increase in REDD1 expression under the influence of metformin, independent of AMPK activation. Similar observations, this time for gliomas, were made by Sesen et al., They also showed a significant increase in Redd1/DDIT4 expression in the case of metformin treatment [50]. This seems to confirm the potential role for this drug in oncological therapy.

### 4.4. Participation in the Caspase 3, BAX and BCL2 Regulation

An important issue in the analysis of the pathogenesis of the neoplastic process is the mechanism of apoptosis regulated by many factors. One example of the pro-apoptic proteins is caspases. The consequence of the disorders of their function has been repeatedly presented in the pathogenesis of gliomas [130,131]. A recent work presented by Xiong et al., has confirmed that metformin can also have an effect on this mechanism. In their work, the authors proved that it could affect the increase in caspase 3 activity in human GBM cells [85]. This suggests the possibility of using the mechanism of metformin-induced apoptosis in cancer therapy. However, the resolution of this issue requires further observations made on animal models and patients with cancer. It is also worth noting that not all GBM studies showed a decrease in the activity of caspases. Zarnescu et al., in their work, showed that the GBM xenografts were characterized by the presence of cells with the increased activity of both caspase 3 and caspase 9. The authors justified this by the fact that some moderate activity of caspases is not sufficient for the massive induction of apoptosis in tumor cells, but contributes to their growth migration capacity [132]. A thorough explanation of this issue, however, requires further research. Other factors affecting apoptosis are pro-apoptic protein Bax and anti-apoptic protein Bcl-2. Tirapeli et al., showed high Bcl-2 activity in glioma cells contributing to the increase of their survival [133]. Sesen et al., also noted that metformin affects the transformation leading to cell death by lowering Bcl-2 levels and increasing Bax expression [85]. This drug can also interact with other factors (P70S6 kinase, plastid ribosomal protein S6 (S6RP)) involved in the regulation of cell proliferation and death [53,134].

### 4.5. Metformin and Immune Microenvironment of Glioma

Tumor associated macrophages (TAMs) have a major impact on the immunological microenvironment of gliomas. In the case of malignant tumors such as GBM, TAMs adopt anti-inflammatory phenotype resulting in the production of anti-tumor immune response inhibiting cytokines, promoting tumor growth and angiogenesis [135,136,137,138]. This is associated with an unfavorable prognosis, which has been demonstrated, among others, in the case of glioma as well as prostate, bladder, breast, and lung cancers [139,140]. Recently, some researchers have suggested the potential effect of metformin in regulating polarization of TAMs reflecting their phenotype. Chiang et al., demonstrated the ability of this drug to inhibit the change of the TAMs phenotype to anti-inflammatory. This was done by the effect of metformin on the increase in AMPK activity and the regulation of the AMPK-NF-κB axis [139]. Similar conclusions were made by Wang et al., who additionally emphasized the anti-angiogenic effects of the drug by inhibiting VEGF expression [141]. In the analysis of GBM immunology, proteins programmed death-1 (PD-1) and programmed cell death ligand (PD-L1) also play an important role. It is recognized that the disturbances in the expression of these particles increase the aggression and invasiveness of GBM cells in the brain tissue [142]. In their work, Cha et al., showed that metformin could also affect this link in the cancer process. By its effect on AMPK, it causes phosphorylation of the S195 of PD-L1 subunit resulting in abnormal PD-L1 glycosylation. The authors observed reduced levels of PD-L1 in patients with breast cancer treated with metformin [143]. The immunological anti-tumor effects of metformin may also be mediated by other mechanisms such as regulation of T cells activity and differentiation [144,145]. The above data confirm another potential mechanism of anti-cancer activity of metformin, increasing the hope of its use in future cancer therapies. However, there is still a lack of work testing the effect of this drug on the high-grade glioma immunological microenvironment. For this reason, further observations will need to be made before determining the true role of metformin in this cancer group.

## 5. Metformin and Glioma Stem Cells (GSC)

Another potential mechanism of the activity of metformin is the effect on stem cell-like glioma cells, also called Glioma Stem Cells (GSC). This is a specific group of tumor cells characterized by high self-renewal capacity, migration, and resistance to cytostatic [146,147]. It is suggested that it is their presence that, in many tumors, determines the tendency to relapse and the resistance to treatment [146,148,149,150,151]. For this reason, they have become an important aspect of research into new therapies gliomas. Some drugs, such as metformin, have the potential for selective killing of cancer stem cells [51,54,152,153].

AMPK has been shown to affect GSC by mediating the inhibition of Forkhead Box O3 (FOXO3) and Protein kinase B (AKT). The FOXO3-AMPK-dependent mechanism may be involved in the GSC differentiation process [52]. This has been proved in studies conducted by Suayama et al., They observed that the activation of the FOXO3 transcription factor could affect the process of distinction of GSC, causing a change in the direction of their transformation into non-cancer cells [154]. Subsequent studies carried out by Sato et al., on a mouse model have indicated the role of metformin in the process of FOXO3 activation by affecting AMPK, thereby causing a change in the direction of GSC transformation in a non-cancerous course. Such a shift causes the depletion of the tumor stem cell population responsible for the self-renewing nature, resulting in a significant improvement of the survival of mice with glioma [52].

## 6. Impact of Metformin on Treatment Sensitivity

Research on glioma cell lines has shown that some of them are more sensitive to metformin than others. In studies conducted by Sesen et al., wild-type (WT) PTEN cell lines were shown to be more sensitive compared to mutated PTEN lines. WT-PTEN present functional PTEN expression, thus being able to oppose PI3K signaling, leading to the inactivation or modulation of the AKT survival pathway. In contrast, mutated PTEN lines show constant PI3K/Akt pathway activity [50]. It has been shown that metformin decreases Akt action in breast cancer cells and in GSC [54,155,156]. However, this interaction is only significant in wildtype cell lines because mutated lines, due to the PI3K/Akt constant activity, are insensitive to modulating factors [50]. This shows that the sensitivity of the tumor to treatment can be determined based on the PTEN status.

The basis of GBM treatment is currently combined therapy in the form of radiation and chemotherapy [8]. The most effective drugs include Temozolomide (TMZ) [157]. It is a derivative of decarbosin, which has been used in the treatment of malignant gliomas for many years. The cytotoxic effect of its action is based on the methylation of the guanine bases in DNA to O-6-methylguanine forms. This causes mispairing and disruption in the process of DNA replication leading to tumor cell apoptosis [158]. However, the mechanism of action of the drug on tumor cells is very complex and involves many signaling pathways. These include the activation of adenosine monophosphate-activated protein kinase (AMPK) [157]. This indicates the potential role of increasing AMPK activity in the effectiveness of temozolomide [159,160]. This effect could be achieved by using metformin.

Metformin has been shown to increase the sensitivity of tumors to radio and chemotherapy [161]. This also applies to combined treatments with TMZ [39,162,163].

The study of Sesena et al., was conducted on glioma cell lines, and showed that co-administration of metformin during therapy with temozolomide enhances its cytotoxicity, the manifestation of which was higher mortality of cancer cells. A similar effect was obtained during simultaneous radiation therapy [50]. Studies carried out by Soritau et al., on tumor cells isolated from patients with high-grade gliomas also showed a significant decrease in the proliferation of neoplasm in therapy with the combination of metformin and TMZ compared to the group of patients treated only with TMZ [164]. A similar effect was seen in the observations of Yu et al. [56]. This demonstrates the potential use of metformin in increasing the effectiveness of the standard types of treatment for highly advanced gliomas in the form of temozolomide and radiation therapy.

Additionally, Valtora et al., investigated the effect of adding metformin to TMZ in observations on GBM cell lines. The authors used both TMZ sensitive cell lines and those resistant to the drug. As expected, monotherapy, using TMZ alone, only had a therapeutic effect on chemosensitive cells. The addition of metformin resulted in a decrease in cell survival for both samples. This seems to confirm the role of metformin in sensitizing tumor cells to TMZ and indicates the potential use of this drug in new glioma therapies. Interestingly, observations of the mouse model showed similar efficacy of metformin alone as in combination therapy, except that the effect was limited only to the first days of treatment. Researchers saw the causes of the anti-tumor effect of both drugs in affecting cancer cell apoptosis. Combination therapy increased apoptosis through reduced Reactive Oxygen Species (ROS) production and increase in Bax/Bcl-2 ratio [165]. As previously mentioned, a variety of factors can influence the characteristics of the tumor and the response of cells to treatment. One of them is the onset of hypoxia. It can cause changes in the tumor phenotype to be more malignant, which is manifested, among others, by an increase in Bcl-2 and Hypoxia-inducible factor 1-alpha (HIF-1α) activity [84]. HIF (hypoxia inducible factor) are transcription factors that react to changes in oxygen concentration in the tumor environment, which under hypoxia induce vessegenesis to ensure adequate oxygen supply in cells [166,167]. They can also affect the sensitivity of the cancer to the therapy [168,169,170]. In observations made by Lo Dico et al., the role of HIF-1α decline in predicting the therapeutic effect of TMZ has been pointed out [171]. In subsequent studies, the authors analyzed the effect of TMZ treatment in combination with metformin or BEZ235 (which is PI3K/mTOR blocker) under hypoxic conditions. The studies were carried out on GBM cell lines, including both TMZ sensitive lines and those resistant to the effects of the drug. Researchers have shown that in hypoxic conditions, combined therapy with TMZ and metformin reduced the survival of cancer cells. The suggested cause was the effect of drugs on the activity of Akt pathway, HIF-1α and VEGF. However, in the case of TMZ resistant cell lines this reaction was limited to HIF-1α only. Combined therapy, also taking into account BEZ235, resulted in a more pronounced anti-tumor effect in resistant lines [171]. This suggests an important role of the PI3K/AKT/mTOR axis in the induction of chemoresistance under hypoxia and confirms that the effectiveness of the therapy combined with the use of metformin also depends on the conditions of the tumor microenvironment.

TMZ activity through methylation of guanine to O-6-methylguanine is associated with the resistance of some types of gliomas. The enzyme O6-methylguanine methyltransferase (MGMT) removes the alkyl groups made by TMZ, causing the repair of its damage and reversing the effect of the drug. This results in low sensitivity of some GBM patients to this type of therapy [158]. Sesen et al., conducted a study on GBM cell lines examining the effect of the combination of TMZ and metformin on proliferation intensity. In their observations, they included both cell lines showing MGMT methylation as well as those in which MGMT was unmethylated. As expected, after using only TMZ in lines where MGMT function was not affected, drug resistance developed. Combination therapy with metformin showed a decrease in proliferation in all samples. A particular difference was seen in resistant lines characterized by the lack of MGMT methylation [50]. Similar results have been shown in studies by Adeberg et al., It is worth emphasizing that, in this experiment, the authors studied the effect of the drug on both cell lines undergoing chemotherapy and radiotherapy. The addition of metformin allowed for reduced cell survival in samples treated with TMZ and radiation therapy alone, as well as in the combination of both treatments. This applied to both methylated MGMT cell lines and those where MGMT remained unmethylated, with greater improvement in the second case [172]. The above studies suggest that this drug may affect the repair processes that determine TMZ resistance and radiation therapy. However, a thorough understanding of the mechanism of this relationship requires further research. Accurate knowledge of this issue may allow for more effective treatment of resistant cases of GBM by adding metformin to the basic treatment.

The possibility of using metformin in combination therapy with other drugs is also being tested. In 2019, Kolesnik et al., conducted a study on the effect of including metformin in cancer therapy using sodium dichloroacetate (DCA). Observations were made on GBM cell lines in vitro and in vivo using a rat model [173]. DCA is a drug that has been mainly used in the treatment of metabolic disorders for many years, but recently its antitumor properties have been studied, also regarding gliomas [174,175,176]. Kolesnik et al., showed that DCA and metformin combined therapy is more effective in reducing the viability of cancer cells by about 40% compared to monotherapy. There was also a clear decrease in glucose consumption by cancer cells (four times less consumption). This affected the life expectancy of rats with intracranial transplanted glioma cells. Combined therapy allowed for the increase in the average life span by 50% compared to the control sample. The antitumor effect was mainly due to the effect on the induction of apoptosis and the cell cycle course of the samples tested. It is worth noting that achieving a therapeutic effect in the case of combined treatment was associated with the administration of smaller doses of the drugs, which is particularly important in light of the threatening side effects [173]. Similar results of simultaneous administration of both drugs were previously seen in observations on ovarian cancer cells [177].

Metformin may also potentiate the effects of other medicines. Aldea et al., made observations of GSC cell lines characterized by resistance to standard treatment. They showed that the combination of metformin and Soraf (RAS/RAF/MAPK pathway inhibitor) results in significantly greater treatment effectiveness manifested in a decrease in TMZ-resistant GSCs proliferation. Both drugs also showed synergism in the production of oxygen free radicals [178]. Similar effects of metformin pro-oxidant have also been reported in the case of the potential for radiation therapy and TMZ [153,157].

It is also suggested that GBM cells have significantly increased the activity of the phosphatidylinositol-3-kinase (PI3K)-Akt pathway and endogenous Akt kinase, in response to the use of TMZ [179,180,181,182,183]. Activation of Akt leads to greater aggressiveness and invasiveness of the tumor [181]. It has also been shown that the increase in Akt expression correlates with the degree of tumor resistance to Temozolomide treatment [179,180,181,182,183]. It was confirmed that GSC cells are highly sensitive to an inhibitor of Akt [180,184]. Furthermore, inhibition of PI3K/Akt pathway signaling causes an increase in the toxic effect of temozolomide [185]. As mentioned earlier, studies on breast cancer cells and GSC suggest that metformin decreases AKT activity [54,155,156]. This indicates another potential metformin pathway and its effect on TMZ activity.

Other biguanide drugs may also have similar properties. In their work, Wang et al., compared the effects of metformin and phenformin on the growth and migration in glioma cell lines. They showed that both drugs are effective in inhibiting tumor cell proliferation. Furthermore, migration ability was also inhibited by affecting Vimentin and E-cadherin expression. A large role in anti-cancer activity has been assigned to an increased level of ROS (Reactive Oxygen Species). In the study by Wang et al., the effectiveness of both drugs was similar [186]. Interestingly, in the previous works describing the effect of these drugs in the treatment of breast cancer, phenformin showed significantly more pronounced activity [187]. However, to date, relatively few papers have been written describing the effect of phenformin on the pathogenesis of gliomas. Jiang et al., have shown that this drug can reduce self-renewal ability in GSC and sensitize them to TMZ [188]. A thorough understanding of the phenformin characteristics in light of the anti-tumor effect requires further research.

## 7. Potentiating Metformin’s Anti-Tumor Effects

Metformin is a relatively safe drug with good tolerance in most patients and a small range of side effects. These usually concern the gastrointestinal system [189,190]. Lactic acidosis is one of the most serious conditions associated with its use, but it is a rare phenomenon, mainly in patients with concomitant organ damage [191,192]. The risk of side effects, including lactic acidosis, increases in high doses of metformin [193], and it is small with standard antidiabetic treatment. In this case, the dose is usually in the range of 500–2500 mg metformin per day [194]. This allows the drug concentration in cerebrospinal fluid and portal vein to reach 40 µM, while the concentration in the brain tissue reaches 10 µM [4,65,195,196]. While some observations indicate that doses of 50–100 µM are sufficient to inhibit respiration at the cellular level in hepatocytes, in the case of low drug levels, the anti-tumor effect is often not particularly pronounced [14,54,90,197]. In most studies reporting the anti-tumor effects of metformin, the doses used were higher than in the standard antidiabetic treatment [8,35,39,51,52,54,55,198]. This puts patients at greater risk of side effects, including lactic acidosis. For this reason, research is being conducted into cumulative therapies that reduce the metformin dose while maintaining anti-cancer efficacy.

Diclofenac is one of the substances capable of potentiating the anti-tumor effect of metformin. It belongs to the group of non-steroidal anti-inflammatory drugs (NSAID), which, due to their effect on cyclooxygenase enzymes (COX-1 and/or COX-2), are used in analgesic and anti-inflammatory therapy [199]. Drugs that inhibit COX-2 activity have also been shown to reduce the risk of developing cancers associated with chronic inflammation [200,201,202]. However, observations made in recent years show that the anti-tumor effect of NSAIDs occurs through both mechanisms dependent and independent of COX [200,203,204,205,206,207,208,209,210]. Moreover, in the recent years, it has been noted that diclofenac also has the ability to inhibit the glycolysis of cancer cells [211,212]. This is very important because of the Warburg effect, which is the rule described in 1956, outlining that cancer cells (also gliomas) obtain the energy needed for tumor growth primarily through the impact on the severity of glycolysis regardless of oxygen demand [213,214,215,216,217]. Inhibiting this process allows them to be cut off from the energy supply needed for tumor growth, which can be used in anticancer therapies [211,218,219,220,221]. It is also suggested that diclofenac contributes to inhibiting lactate transport in cells whose continuous outflow is necessary to maintain adequate proton concentration gradient and ATP production. Proper functioning of this process is needed for tumor growth, and its disturbances affect cancer cells more than normal ones [222,223,224,225,226].

The combination of the effects of metformin and anti-inflammatory drugs, by affecting the mechanism of glucose transformation in cancer cells, allows their anti-tumor activity to accumulate and to apply effective therapy using lower doses of drugs. In a study conducted by Saber et al., combination therapy of metformin with 5-aminosalicylacid has been shown to be associated with the inhibition of proliferation in colorectal cancer cell lines [227]. Similar observations were made by Gerthofer et al., on glioma cell lines. They showed that therapy in the form of simultaneous administration of diclofenac and metformin causes a decrease in the severity of glycolysis and the outflow of lactate from cells. The proliferation and migration of cancer cells was also inhibited. This effect was greater with concomitant medication compared to the treatment with metformin alone [4]. This shows a potential role for diclofenac in modern GBM therapies.

2-Deoxyglucose (2DG) appears to be another substance that could potentiate the effects of metformin. It is a modified glucose molecule in which the 2-hydroxyl group has been replaced by hydrogen. The effect of this is a blocked glycolysis pathway [216,228,229,230,231]. As mentioned earlier, due to the Warburg effect, it particularly affects tumor cells [213,215]. The antitumor impact of 2-deoxyglucose has been shown in breast and pancreatic cancer [219,232] based on its cumulative effect of the co-administration of metformin. Such observations were made in the case of prostate cancer and pancreatic cancer [220,232,233,234]. The potentially beneficial effect of combining these two drugs was suggested in studies conducted by Kennedy et al. [234]. This was confirmed by the observations made by Kim et al., on GBM-derived tumorspheres (TS) and mouse orthotopic xenograft model. They showed that the simultaneous administration of both drugs was associated with the stronger inhibition of tumor cell growth compared to the control. The effect on energy metabolism has been confirmed by measuring ATP levels [197]. Interestingly, the use of only metformin was associated with a weak response in the form of inhibition of GBM-TS proliferation in both its low (5 mM) and high (15 mM) concentration. Furthermore, both metformin and 2-deoxyglucose have the ability to cross the blood-brain barrier, which underlines their potential importance in the treatment of gliomas [85,235].

Trials are currently underway with many other drugs with potential cumulative effects with biguanide derivatives. This group includes, among others: Sorafenib, Cisplastin, Gemcitabine, Gefitinib, Fluorouracil, and 9-cis retinoic acid (9-cis RA) [43,85,178,236,237,238]. Further research in this field may allow for more effective anti-cancer treatment while minimizing the side effects of the therapy experienced by the patient.

## 8. Effect of Metformin on the Pathogenetic Mechanism of Brain Edema

Metformin may not only affect the dynamics of cancer development but also the severity of its symptoms. Recent observations have shown its effect on brain edema. The presence of a tumor in the vast majority of cases is associated with swelling of the surrounding tissue. This is done through various mechanisms. For this reason, edema due to a cancer is divided into:

- vasogenic edema, and
- cytoxic edema [239,240].

Brain-blood barriers (BBB) is a physical and biochemical boundary between blood vessels and nerve tissue [241]. Its essence is to regulate the selective transport of substances between blood and cerebrospinal fluid, as well as to protect the nervous system against the influence of harmful factors from the bloodstream [242]. BBB consists of endothelial cells connected through tight junctions also called occluding junctions or zonulae occludentes. They are protein complexes whose function is to create a border between tissues to prevent leaks of water and substances dissolved in it [243,244,245]. Tight junctions incorporate over 40 proteins, including Claudin family, Ocludins and junction adhesion molecules (JAM) [246,247]. Many authors underline their role in BBB’s integrity and proper function [248,249]. Loss of proteins from this group may result in brain edema [250].

The presence of brain tumors often causes disorders of the blood-brain barrier due to reduced expression of tight junctions. Numerous observations indicated that this could be due to hypoxia caused by the presence of rapidly growing cancer cells resulting in increased levels of vascular endothelial growth factor (VEGF) [239,240,251,252].

The beneficial effects of metformin on the blood-brain barrier have been demonstrated in studies by Takata et al., They checked the effect of its presence on rat brain microvascular endothelial cells (RBECs). The state of the blood-brain barrier was determined by the effect of metformin on transendothelial electrical resistance (TEER) and permeability of sodium fluorescein (NaF) of the cells tested. They showed that adding the drug causes an increase in TEER and a decrease in NaF, reflecting the proper barrier tightness. This mechanism was probably dependent on the effect of metformin on AMPK activity [253].

In addition, the researchers noted a positive response after the administration of the drug to the expression level of zona occuldens protein 1 (ZO-1) and occludin. This is a very important fact because the previous work indicated their key role in the correct barrier structure [254]. ZO-1 is a protein classified as membrane-associated guanylate kinase-like protein. It contains guanyl kinase like domain which, through the reaction with occluding, is involved in the formation of the protein scaffold necessary to maintain transmembrane proteins in the cytoskeleton of epithelial cells, having great importance for the proper function of BBB [255,256,257,258]. In addition, disorders of ZO-1 and Occludin levels have been repeatedly observed in pathological conditions accompanied by cerebral edema [257,259,260,261].

Another mechanism that may affect the development of brain edema is the level of expression of aquaporin-4 protein (AQP4). It is a protein belonging to the aquaporin family, which is the most common aquaporin channel in CNS [262,263,264]. Its increased expression concerns the structure of the blood-brain barrier [265]. Moreover, the results of studies carried out by Toma-sCamardiel et al., suggest that it correlates well with the level of BBB permeability [266]. This is confirmed by other studies in the light of which there is a relationship between the amount of AQP4 and the severity of tumor-induced brain edema [267,268].

The beneficial effect of metformin on reducing astrocyte edema in vitro has been observed to date [269]. Research conducted by Zhao et al., on astrocyte cultures has proved that metformin can lower AQP4 levels. It has been suggested that its effect on increased AMPK activation and attenuated NF-κB activation is responsible for this. The same researchers demonstrated a significant reduction in brain edema and glioma induced vascular permeability [252]. This seems to confirm the multidimensional effect of this drug on the pathomechanism of edema.

Metformin can also reduce the adverse effects of inflammation on endothelial cells, which are part of the blood-brain barrier. This is done by inhibiting TNF-α (tumor necrosis factor-α)-induced IL-6 production associated with PI3K-dependent AMPK phosphorylation [270]. This effect may potentially be beneficial for the function of the blood-brain barrier, but further studies are necessary to thoroughly understand the nature of this relationship.

## 9. Conclusions

Recent years have brought many observations showing metformin in its new role. The drug commonly used in the therapy of diabetes may find application in the therapy of many tumors. Its effectiveness has been demonstrated in colon, breast, prostate, and pancreatic cancer, leukemia, melanoma, lung and endometrial carcinoma, as well as in gliomas. This is important because, in the case of high-grade gliomas, which include GBM, the therapeutic options available to patients are poor. Research suggests the possibility of increasing the effectiveness of standard therapies, which include radiation and chemotherapy, usually with temozolomide. In many cases, this can make it possible to inhibit tumor growth, limit relapses, and consequently, save many patients. Despite the beneficial effects on tumor metabolism, the exact mechanism of action of metformin remains a mystery. Past observations suggest the involvement of signaling pathways including AMPK, mTOR, REDD1, and mechanisms regulating cellular respiration and oxidative stress. However, many of the studies presented in this paper have been carried out on glioma cell lines. Despite some very interesting conclusions, such models do not offer the full picture of the complexity of the tumor process and should be complemented by appropriate experiments performed on animal models or observations of patients. It shows that, in order to know the exact characteristics of metformin’s effect on high-grade glioma metabolism, further studies will be needed. A thorough understanding of the mechanism of this medication can make it possible to discover new drugs that can be used in neoplasm therapy.

## Figures and Tables

**Table 1:** Currently registered clinical trials considering the use of metformin in glioma and cerebral tumors treatment.

No.	Trial	Start Date	Estimate Completiton Date	Country	*n*	Title
1	NCT01430351	September 2011	September 2022	USA	144	Temozolomide, Memantine Hydrochloride, Mefloquine, and Metformin Hydrochloride in Treating Patients With Glioblastoma Multiforme After Radiation Therapy
2	NCT02496741	November 2015	December 2016	Netherland	20	Metformin And Chloroquine in IDH1/2-mutated Solid Tumors
3	NCT02040376	March 2014	December 2017	Canada	24	Placebo Controlled Double Blind Crossover Trial of Metformin for Brain Repair in Children With Cranial-Spinal Radiation for Medulloblastoma
4	NCT02149459	June 2014	July 2018	Israel	18	Treatment of Recurrent Brain Tumors: Metabolic Manipulation Combined With Radiotherapy (SMC 0712-13)
5	NCT02780024	March 2015	December 2020	Canada	50	Metformin, Neo-adjuvant Temozolomide and Hypo- Accelerated Radiotherapy Followed by Adjuvant TMZ in Patients With GBM
6	NCT03243851	November 2016	December 2019	Korea	108	Study on Low Dose Temozolomide Plus Metformin or Placebo in Patient With Recurrent or Refractory Glioblastoma (METT)
7	NCT03151772	January 2018	March 2021	Sweden	40	Bioavailability of Disulfiram and Metformin in Glioblastomas
